# High Prevalence of Multi-Viral Co-Infections and Low Rabies Seropositivity in Stray Cats of Shenzhen, China

**DOI:** 10.3390/ani15203042

**Published:** 2025-10-20

**Authors:** Tinglu Wang, Mengmeng He, Yan Liu, Runchang Lin, Rongjie Huang, Bowen Lin, Yinyi Liang, Xiaofeng Guo, Rongqi Liu, Jun Luo

**Affiliations:** 1 College of Veterinary Medicine, South China Agricultural University, Guangzhou 510642, China; wangtinglu1999@163.com (T.W.); Liuyannni@163.com (Y.L.); rjhuang@stu.scau.edu.cn (R.H.); bobysmail@163.com (Y.L.); xfguo@scau.edu.cn (X.G.); 2 Shenzhen Inspection and Testing Center of Agricultural Product Quality and Safety, Shenzhen 518000, China; m_m_he@163.com (M.H.); sz1322755037@163.com (R.L.); wongojack@163.com (B.L.); 3 Zhaoqing Branch Center of Guangdong Laboratory for Lingnan Modern Agricultural Science and Technology, Zhaoqing 526000, China

**Keywords:** stray cats, feline coronavirus type I (FCoV-I), feline calicivirus (FCV), feline herpesvirus type I (FHV-I), feline panleukopenia virus (FPV), rabies virus (RABV)

## Abstract

**Simple Summary:**

Stray cats (*Felis vaga*) can carry and spread diseases that affect both other cats and sometimes people. In this study, we wanted to find out how common certain important viruses are among stray cats in Shenzhen, China. We also checked if the cats had protection against rabies, a serious disease that can be passed to humans. We collected samples from 126 stray cats between June and August 2024. Our tests showed that these viruses were very common overall. Most cats were infected with at least one virus, and many were infected with more than one at the same time. We did not find the rabies virus in any cat. However, very few cats had antibodies against rabies, meaning they were not protected from the disease and could potentially spread it if infected. This study tells us that stray cats in Shenzhen commonly carry several cat viruses and have low immunity to rabies. This information is important for planning strategies to manage the stray cat population and to protect both cat and human health.

**Abstract:**

Stray cats (*Felis vaga*) are key hosts for feline and zoonotic pathogens. From June to August 2024, we conducted a cross-sectional study across six districts in Shenzhen, China, involving 126 cats sampled from three types of sites. Multiple specimens were tested via quantitative real-time PCR (qPCR) for feline coronavirus type I (FCoV-I), feline calicivirus (FCV), feline herpesvirus type I (FHV-I), feline panleukopenia virus (FPV), and rabies virus (RABV); serum was analyzed for RABV-neutralizing antibodies by the fluorescent antibody virus neutralization (FAVN) assay. The overall pathogen positivity was 89.68%. FPV was most prevalent (61.90%), followed by FCV (57.14%), FCoV-I (46.83%), and FHV-I (23.02%). No RABV nucleic acid was detected. The co-infection rate reached 62.70%, primarily dual infections (33.33%). Geographical variation was observed, with significantly higher FCoV-I in Longgang than Futian (*p* < 0.05). RABV seropositivity was only 6.00%. FCV and FPV co-occurred most frequently (Jaccard = 0.456). All pathogen pairs had relative risk (RR) > 1, suggesting non-random co-infections, though not significant after correction. In summary, major feline pathogens are widespread with frequent co-infections among Shenzhen stray cats, while low rabies immunity indicates potential public health risk. Targeted control measures are warranted.

## 1. Introduction

In recent years, the growth rate of the domestic cat *(Felis catus)* population in China has exceeded that of domestic dogs *(Canis lupus familiaris)*, with the total number of cats approaching that of dogs, particularly in urbanized areas. The expanding cat population has led to an increase in lost, abandoned, and unregulated breeding cases, resulting in a yearly surge in the number of stray cats *(Felis vaga)*. Stray cats can carry infectious pathogens, and due to their mobile nature, they can easily transmit diseases to other healthy stray cats and even household cats, which could potentially trigger large-scale outbreaks. This situation also poses a risk of zoonotic disease transmission. Therefore, the prevention and control of major diseases in urban stray animals are of critical importance.

Common pathogens affecting cats include feline coronavirus (FCoV), feline calicivirus (FCV), feline herpesvirus (FHV), feline panleukopenia virus (FPV), and rabies virus (RABV). FCoV, which belongs to the *Coronaviridae* family, possesses a positive-sense single-stranded RNA genome. It is divided into two genotypes: type I and type II. Type II feline coronavirus (FCoV-II) originated from recombination between type I feline enteric coronavirus (FECV) and canine coronavirus (CCoV) [1]. In natural infections, type I feline coronavirus (FCoV-I) is the predominant form [2,3]. Based on pathogenicity, FCoVs are categorized into two biotypes: the mildly pathogenic strains causing mild or subclinical gastrointestinal symptoms (Feline enteric coronavirus, FECV) and the highly lethal strains (Feline infectious peritonitis virus, FIPV), which arise from spontaneous mutations of FECV [3,4]. FCV, a member of the *Caliciviridae* family, possesses a positive-sense single-stranded RNA genome. It is highly contagious and primarily causes oral ulcers, mild upper respiratory symptoms, and can lead to severe pneumonia in kittens. While kittens exhibit high morbidity and mortality rates, adult cats often remain asymptomatic; however, they can become virus carriers [5]. FHV-I, an α-herpesvirus with a double-stranded DNA genome, is the primary etiological agent of feline viral rhinotracheitis, accounting for approximately half of all feline upper respiratory infections. Clinical signs include conjunctivitis, epiphora, nasal discharge, coughing, sneezing, and anorexia. Although cats of all ages are susceptible, kittens between 2 and 4 months of age are most vulnerable, exhibiting an incidence rate of 100% and a mortality rate of up to 50%. The virus is transmitted through direct contact, fomites, and aerosol droplets, with documented potential for cross-species transmission to chinchillas *(Chinchilla lanigera)* [6]. A hallmark of FHV-1 infection is the establishment of latency and subsequent intermittent viral shedding, during which infected cats can periodically excrete infectious virus [7]. FPV, a member of the *Parvoviridae* family, possesses a single-stranded DNA genome. It is the causative agent of feline panleukopenia, also known as feline infectious enteritis (FIE). Intrauterine or neonatal infection can result in cerebellar hypoplasia, with reported mortality rates ranging from 25% to 100% [8]. FPV exhibits high resistance to common physical and chemical factors. The virus is transmitted via the fecal-oral route, either through direct contact or indirect environmental contamination, facilitating outbreaks in feral cat populations. Consequently, effective vaccination and rigorous disinfection are essential for controlling FPV spread in animal shelters. RABV, the agent of a lethal zoonosis, can infect almost all warm-blooded animals and is responsible for approximately 60,000 human deaths annually [9]. The predatory and nocturnal behavior of cats increases their exposure to bats (Chiroptera), a key reservoir, while their close contact with humans as companion animals heightens the risk of zoonotic transmission.

In this study, for the first time, we tested 126 stray cats sampled from six urban districts of Shenzhen (Bao’an, Nanshan, Luohu, Longgang, Longhua, and Futian) for major feline infectious diseases and zoonotic pathogens between June and August 2024. We conducted a comprehensive analysis of the pathogen carriage and transmission risks within this stray cat population. Our findings provide critical data for the prevention and early warning of feline infectious diseases in Shenzhen, as well as a scientific basis for assessing rabies transmission risks and formulating effective control strategies.

## 2. Materials and Methods

### 2.1. Sample Collection

From June to August 2024, samples were collected from 126 stray cats in six distinct districts of Shenzhen. The target number of cats was determined with reference to sample sizes commonly employed in comparable cross-sectional studies of urban stray cats in China [10]. Three types of sites were targeted to maximise demographic and exposure diversity:

(i) veterinary clinic that serves as a drop-off point for injured free-roaming cats;
(ii) municipal trap–neuter–return (TNR) shelter;
(iii) outdoor feeding site.

At each site, every newly presenting cat that could be safely handled was sampled until the target number was reached. This approach does not constitute random sampling of the entire stray cat population, but it captures a broad spectrum of husbandry conditions and likely exposure histories. Each cat contributed at least one, but not necessarily all, of the following specimen types: whole blood (*n* = 124), serum (*n* = 100), nasal swab (*n* = 116), ophthalmic swab (*n* = 102), oropharyngeal swab (*n* = 126), anal swab (*n* = 109) and feces (*n* = 76). The samples were stored at −20 °C until analysis. Detailed individual records, including body weight, sex, breed, age, and the presence or absence of clinical signs (ocular discharge, stomatitis, cough), are provided in Supplementary Table S1. Because of the fractious nature of stray cats, sample collection was limited to what could be obtained during a single, brief physical restraint; consequently, not every cat yielded all intended specimen types, resulting in an incomplete and variable sampling matrix.

### 2.2. Cells and Viruses

The BHK-21 cells were cultured in DMEM supplemented with 10% fetal bovine serum (FBS). RABV challenge virus standard 11 (CVS-11) strain was propagated and cultured in BHK-21 cells (kindly provided by Dr. Xianzhu Xia, Academy of Military Medical Sciences, Beijing, China).

### 2.3. Sample Treatment

Swabs (eye, nasal, oral, and anal) were placed in centrifuge tubes with 1 mL of phosphate-buffered saline (PBS) and vortexed to dissolve the samples. For whole blood, 200 μL of anticoagulant-treated blood was added to the buffer and mixed thoroughly. Non-anticoagulated blood was centrifuged at 3000 rpm for 10 min to collect serum.

### 2.4. Nucleic Acid Extraction

Viral DNA/RNA was extracted using the Virus DNA/RNA Nucleic Acid Extraction Kit 2.0 (Vazyme Biotech, Nanjing, China) on the VNP-32P fully automated nucleic acid extraction system (Vazyme Biotech, Nanjing, China). The eluates were stored at −80 °C.

### 2.5. Fluorescence Quantitative PCR (qPCR)

Pathogen detection (FPV, FCoV-I, FHV-I, FCV, and RABV) was performed using specific Viral Nucleic Acid Test Kits (Shenzhen Lvshiyuan Biotechnology, Shenzhen, China) on the CFX Connect Real-Time System (Bio-Rad, Hercules, CA, USA) and QuantStudio™ Real-Time PCR System (Thermo Fisher Scientific, Waltham, MA, USA).

### 2.6. Result Determination of qPCR

- Quality control: The negative control is FAM channel without amplification curve, Ct value shows no value or Ct value = 40. The positive control showed amplification curves in the FAM channel with Ct values ≤ 35. The above two requirements must be met simultaneously in the same experiment; otherwise, this experiment is invalid.
- Interpretation of test results: When there is an amplification curve in the FAM detection channel of the sample and the Ct value is ≤36, it can be interpreted as pathogen positive. When there is no amplification curve in the FAM detection channel of the sample and the Ct value shows no value or Ct value = 40, the result is judged as pathogen negative. When the Ct value of the sample is greater than 36 and less than 40, it is considered a gray area, and the experiment needs to be repeated. When the Ct value of the retest result is less than 40, the corresponding item of the detection channel is considered positive, otherwise it is considered negative.

### 2.7. Fluorescent Antibody Virus Neutralization Test (FAVN)

FAVN was conducted with CVS-11 to determine the virus neutralizing antibodies (VNA) of RABV in the serum as described previously [11]. Briefly, The BHK-21cells were cultured in DMEM with 10% FBS. The serum was inactivated at 56 °C for 30 min and stored at −20 °C for use. Take a 96-well cell culture plate with 4 rows × 8 columns as a sample detection area, and set up a standard plate, divided into 5 zones: standard reference serum (0.5 IU/mL) area (4 rows × 8 columns), the standard serum positive control (1 IU/mL) area (4 rows × 8 columns), the viral titer detection area (4 rows × 6 columns), the negative serum area (4 rows × 1 column), and the cell control area (4 rows × 1 column). A total of 150 µL of Dulbecco’s Modified Eagle Medium (DMEM) was added to each well in the virus detection zone, and 100 µL was added to the remaining partitions. A standard serum (WHO) of 0.5 IU/mL was used as the standard reference serum, 1 IU/mL of standard serum will be used as the positive control, and serum without rabies virus antibody will be used as the negative serum. Add 50 µL of standard reference serum to each of the 4 wells in column 1 of the standard serum reference area, mix thoroughly with a multichannel pipette, and make a 3-fold gradient dilution. The dilution of positive control, serum to be tested and negative serum zone is the same as above. The CVS-11 strain was diluted with DMEM to make the final titer of virus 100 TCID_50_/50 µL. After mixing thoroughly, add the above virus dilution into the wells of column 1 of the viral titer assay zone, and then dilute it by 4-fold gradient. Add 50 µL of virus dilution to each well of the other zones and incubate at 37 °C for 60 min. After the neutralization reaction, add 50 µL of trypsin-digested BHK-21 cell suspension to each well, and finally incubate the 96-well plate in an incubator at 37 °C with 5% CO_2_ for about 48 h. Remove the cell culture plate, discard the cell culture medium, add 150 µL of pre-cooled 80% acetone solution to each cell well, and incubate for 40 min at −20 °C. discard acetone, and wash the cells with PBS (0.01 mol/L, pH = 7.6) for 3 times. Add 50 µL of FITC-labeled anti-RABV nucleoprotein (N) antibody (Fujirabio Diagnostics, Inc., East Whiteland, PA, USA) to each well and incubate overnight at 4 °C under light. The fluorescent antibody was discarded and the cells were washed 3 times with PBS (0.01 mol/L, pH = 7.6), dried naturally, and visualized under a fluorescence microscope (AMG).

### 2.8. Statistical Analysis

Data from stray cats were organized and statistically analyzed using Microsoft Excel 2019. The heatmap illustrating co-infection patterns based on the Jaccard index was generated using GraphPad Prism 10. The co-occurrence network of pathogens based on relative risk (RR) was constructed with RAWGraphs 2.0. The geographical distribution of pathogen infection rates across different administrative districts of Shenzhen was visualized using Datawrapper.

The significance of differences in the prevalence of the four pathogens across the six administrative districts was assessed using Fisher’s exact test in GraphPad Prism 10. Post hoc pairwise comparisons were performed with Bonferroni correction in Excel 2019 (correction factor k = 15). Associations for pairwise co-infections among the four pathogens were also evaluated using Fisher’s exact test in GraphPad Prism 10, and *p*-values were adjusted with Bonferroni correction in Excel 2019 (correction factor k = 6). The 95% confidence intervals (95% CI) for positive rates were calculated using the Wilson score method with asymmetrical continuity correction, a recommended approach for binomial proportion data. This was implemented with the VassarStats online tool (http://vassarstats.net/prop1.html (accessed on 11 October 2025)).

The odds ratio (OR) for each pathogen pair was calculated using GraphPad Prism 10. Relative risk (RR) and the Jaccard similarity coefficient were computed in Excel 2019 using the following formulas:

The relative risk (RR) was calculated as:

$$\mathrm{RR}\;=\;\frac{O_{AB}}{E_{AB}}\;=\;\frac{a/N}{\left[\left(a+b)\times (a+c\right)\right]/N^{2}}$$

where $O_{AB}=a/N$ represents the observed frequency of co-infection, and $E_{AB}=\frac{\left(a+b\right)\;\times \;(a+c)}{N^{2}}$ denotes the expected frequency of co-infection under the assumption of independence. Here, a indicates the number of samples positive for both pathogens, b represents samples positive only for pathogen A, c represents samples positive only for pathogen B, d represents samples negative for both pathogens, and N is the total sample size.

The Jaccard similarity coefficient was calculated as:

$$J=\frac{a}{a+b+c}$$

where a denotes the number of samples positive for both pathogens, b represents samples positive only for pathogen A, and c represents samples positive only for pathogen B.

## 3. Results

### 3.1. Sample Information and Overall Prevalence

A total of 126 stray cats from six administrative districts in Shenzhen (Futian, Luohu, Longgang, Nanshan, Bao’an, and Longhua) were included in this study. The data presented herein are based on the individual cat as the unit of analysis. A cat was considered positive for a pathogen if the pooled swab sample tested positive by qPCR. The sample size from each district exhibited an uneven distribution due to sampling constraints. Nucleic acid testing was performed for the following pathogens: FCOV-I, FCV, FHV-I, FPV, and RABV. As shown in Table 1, 113 out of the 126 cats tested positive for at least one pathogen, yielding an overall positivity rate of 89.68% (95% CI: 83.14–93.87). Among the detected pathogens, FPV showed the highest prevalence at 61.90% (95% CI: 53.19–69.91), followed by FCV at 57.14% (95% CI: 48.41–65.44), FCOV-I at 46.83% (95% CI: 38.34–55.51), and FHV-I at 23.02% (95% CI: 16.53–31.10). No positive cases were detected for RABV, with an estimated upper limit of prevalence of 2.96% (95% CI). The cycle threshold (Ct) values for all positive detections across the different specimen types are summarized in Supplementary Table S2, providing an overview of the pathogen load distribution.

### 3.2. Analysis of Pathogen Co-Infection Patterns

Out of the 126 cats studied, 79 were found to have mixed infections (Table 2), with a mixed infection rate of 62.70% (79/126). Among them, 42 cats were mixed with two pathogens, accounting for 33.33% (42/126), 28 cats were mixed with three pathogens, accounting for 22.22% (28/126), and 9 cats were mixed with four pathogens, accounting for 7.14% (9/126).

Among the 42 cats with dual infections, the co-infection of FCV and FPV was the most prevalent combination, representing 35.71% (15/42) of dual infections and 11.90% (15/126) of the total population. This was followed by the combinations of FCoV-I and FCV, as well as FCoV-I and FPV, each accounting for 26.19% (11/42) of dual infections (and 8.73% of the total population). Furthermore, the combination of FHV-I and FPV accounted for 9.52% (4/42) of dual infections (3.17% of the total). Finally, the combination of FCV and FHV-I was observed in 2.38% (1/42) of dual infection cases (0.79% of the total), while no co-infection of FCoV-I and FHV-I was detected.

Among the 28 cats with triple infections, the most frequent combination was FCoV-I, FCV, and FPV, which constituted 57.14% (16/28) of triple infections (12.70% of the total population). This was followed by the combination of FCV, FHV-I, and FPV, accounting for 25.00% (7/28) of triple infections (5.56% of the total). The combination of FCoV-I, FHV-I, and FPV represented 10.71% (3/28) of triple infection cases (2.38% of the total), and the combination of FCoV-I, FCV, and FHV-I accounted for 7.14% (2/28) of triple infections (1.59% of the total).

All 9 cases of quadruple infections involved FCoV-I, FCV, FHV-I, and FPV, representing 100% of quadruple infection cases (7.14% of the total population).

**Figure 1:**
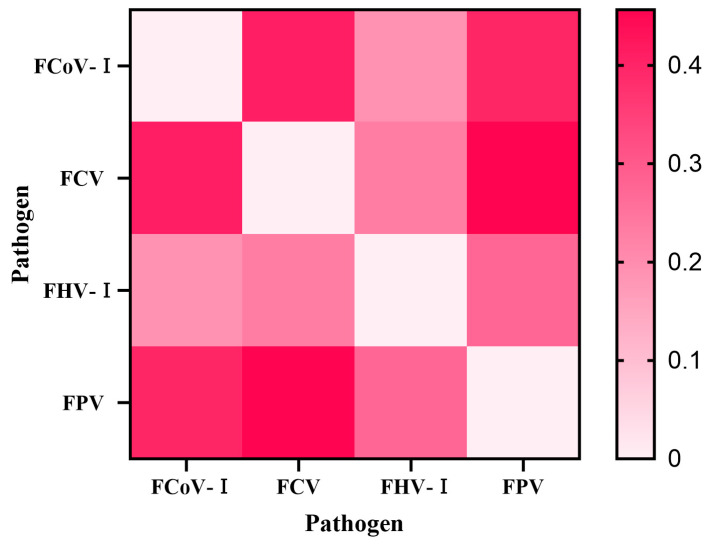
Heatmap of co-infection patterns based on Jaccard index. Each cell represents the Jaccard index value for a pathogen pair, with color intensity proportional to the index magnitude (darker shades indicate higher values). The diagonal represents self-comparison with Jaccard index defined as 0.

**Figure 2:**
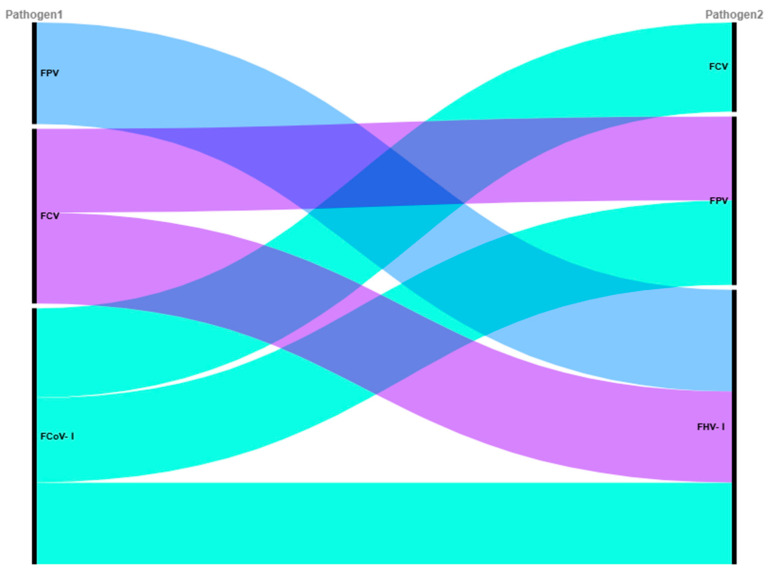
Co-occurrence network of pathogens based on Relative Risk (RR). Nodes represent individual pathogens. Edges connect pathogen pairs with detectable associations, with edge thickness proportional to the magnitude of the RR value. The layout of the network illustrates the overall structure of co-occurrence relationships among the four pathogens.

To further evaluate the pairwise co-occurrence association strength between pathogens, we calculated the relative risk (RR), odds ratio (OR), and Jaccard index (Table 3). The RR values for all pathogen pairs were greater than 1 (range: 1.031–1.281), suggesting a higher observed co-infection rate than would be expected under the assumption of statistical independence. Although the FHV-I-FPV pair had the highest odds ratio (OR = 2.93) and the corresponding Fisher’s exact test yielded a nominally significant *p* value (*p* < 0.05), after Bonferroni multiple testing correction (correction factor k = 6), none of the pairwise pathogen co-infection associations remained statistically significant (adjusted *p* > 0.05). The co-infection heatmap based on the Jaccard index (Figure 1) visually indicated that FCV and FPV had the highest tendency for co-occurrence (Jaccard index = 0.456), while FCoV-I and FHV-I showed the lowest (Jaccard index = 0.189). This pattern was further supported by the co-occurrence network diagram based on RR values (Figure 2), in which the thickness of the edges between nodes (pathogens) represents the magnitude of the RR value.

### 3.3. Geographical Distribution Characteristics of Pathogen Infections

**Figure 3:**
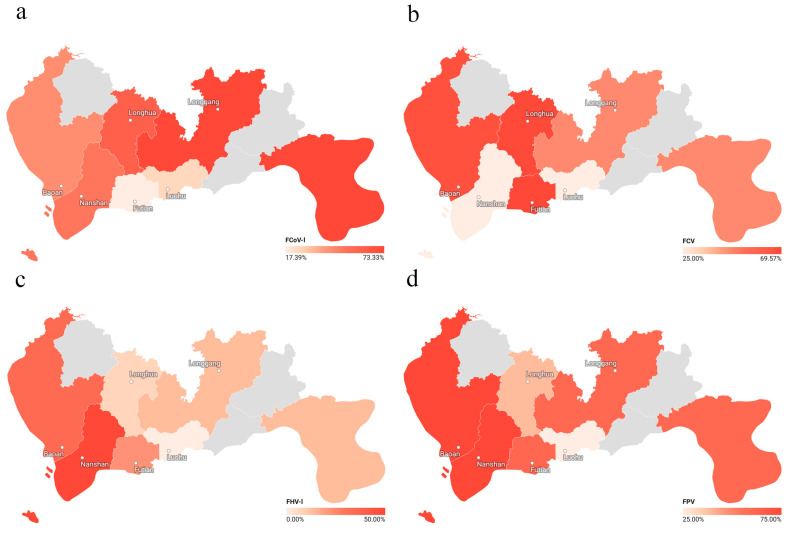
Geographical distribution of pathogen infection rates across Shenzhen. The spatial variation in infection rates for each pathogen within the six administrative districts of Shenzhen is shown: (**a**) feline coronavirus type I (FCoV-I), (**b**) feline calicivirus (FCV), (**c**) feline herpesvirus type I (FHV-I), and (**d**) feline panleukopenia virus (FPV). Each district is visualized using a color gradient representing the level of pathogen positivity, with darker shades indicating higher infection rates. The geospatial visualization was created using Datawrapper by importing the geographical boundaries of Shenzhen and corresponding epidemiological data from each district.

The prevalence rates of the four pathogens exhibited distinct spatial distribution patterns across the six administrative districts of Shenzhen (Figure 3). Geographical correlation analysis revealed globally significant differences in the prevalence rates among regions for all four pathogens (FCoV-I, *p* = 0.0010; FCV, *p* = 0.042; FHV-I, *p* = 0.037; FPV, *p* = 0.043). However, upon post hoc pairwise comparisons with Bonferroni correction, only the difference in the distribution of FCoV-I between Longgang District and Futian District remained statistically significant (adjusted *p* = 0.001), with the infection rate in Longgang being significantly higher than that in Futian (Table 4). The differences in the prevalence of the remaining pathogens among the districts were no longer significant after correction.

In addition, among the six districts surveyed, the positive rate of FCoV-I was the highest in Longgang District, at 73.33% (22/30), FCV was the highest in Futian District, at 69.57% (16/23), FHV-I was the highest in Nanshan District, at 50% (4/8), and FPV was the highest in Bao’an District and Nanshan District, at 75% (33/44) and 75% (6/8), respectively (Table 4).

### 3.4. Patterns of Mixed Infections Across Regions

Among the six districts surveyed in Shenzhen, Nanshan District had the highest mixed infection rate at 75.00%, followed by Bao’an District at 72.73%. Among the six districts, Bao’an and Futian District had the highest number of stray cats infected with FCV and FPV, with positive rates of 15.91% and 26.09%, respectively. Nanshan District had the highest number of stray cats infected with FCoV-I and FPV (25.00%). Luohu District had only one case of mixed infection with FCoV-I and FCV, which was the lowest among the six districts. Longgang District had the highest number of stray cats infected with FCoV-I, FCV and FPV (23.33%). Longhua District had the highest number of stray cats infected with FCoV-I and FCV (38.46%) (Table 5).

### 3.5. Results of Rabies Neutrality Antibody Testing for Stray Cats

Among the 100 serum samples collected from stray cats, 94 were negative for rabies neutralizing antibodies and 6 were positive for rabies neutralizing antibodies, with a positive rate of 6% (6/100) (Table 6). The positive rate of rabies neutralizing antibodies in Nanshan District is the highest (25.00%, 2/8), followed by Longgang District (13.64%, 3/22) and Futian District (5.26%, 1/19). and Bao’an District and Longhua District at 0.00%.

In addition, among 100 serum samples, the positive rate of neutralizing antibodies in samples from the central urban area was 6.06% (4/66), and the positive rate of neutralizing antibodies in peripheral areas was 5.88% (2/34) (Table 7).

## 4. Discussion

Shenzhen, as a densely populated and economically developed city in China, hosts a large population of pet owners. The frequent contact opportunities between humans, pets, and stray cats may elevate the risk of disease transmission, posing a major threat to the health of domestic cats and urban public health. FCoV-I, FCV, FHV-I, and FPV are important pathogens that endanger cats. Given that Shenzhen is located in South China, a region with high rabies incidence, the detection of RABV nucleic acid and neutralizing antibodies in stray cats is highly relevant for rabies prevention and control efforts.

Currently, there are limited surveillance reports on important infectious diseases and zoonotic pathogens in stray cats. In this study, nucleic acids of FCoV-I, FCV, FHV-I, and FPV were detected in the stray cats, while no positive results for RABV nucleic acid were found, and the positive rate of neutralizing antibodies was extremely low. A notable strength of this study is the integration of molecular detection with clinical observations across all 126 cats, which provides valuable insights into the prevalence and clinical significance of these infections among stray cats in Shenzhen.

However, several limitations must be considered when interpreting these findings. First, our sampling strategy did not constitute a random sample of the stray cat population in Shenzhen. Sampling sites were limited to veterinary clinics with stray cat treatment programs, TNR shelters, and community feeding points. Therefore, our cohort may not be fully representative of the overall prevalence rates. Additionally, during sample collection, the high wariness and aggression of some stray cats permitted only brief physical restraint, resulting in the inability to collect all intended samples from every individual. This limitation may introduce selection bias.

Second, a key methodological limitation of the viral detection component is its reliance on molecular methods (qPCR) alone. While highly sensitive for identifying pathogen nucleic acid, a positive qPCR result does not necessarily indicate the presence of live, infectious virus, which limits our assessment of the actual transmission risk. For future studies aiming to characterize active shedding and transmission dynamics, integrating virus isolation with molecular methods, as demonstrated by Acar et al. for FCV [12], would be a crucial advancement. It should be noted that this limitation applies only to the molecular detection of the five viral pathogens; the assessment of rabies virus neutralizing antibodies was conducted using the fluorescent antibody virus neutralization (FAVN) test, which is considered the gold standard.

Despite these limitations, our analysis revealed complex and insightful pathogen-symptom relationships in this population. We confirmed a strong correlation between FCV infection and stomatitis, consistent with the well-documented tropism of calicivirus for oral tissues [13]. Other symptoms, such as diarrhea and dermatopathy, were often associated with mixed infections. Interestingly, contrary to classical expectations, ocular discharge was not definitively associated with FHV-I; some affected cats were FHV-I-negative but positive for other pathogens such as FPV. These findings suggest that while classic patterns (e.g., FCV-stomatitis) remain evident, certain clinical signs like ocular discharge may be caused by other pathogens or non-infectious factors. Collectively, these correlations indicate that a substantial proportion of detected infections were clinically active. The absence of hematological parameters further limited a comprehensive assessment of disease status for some conditions.

FPV is associated with high mortality rate and is taken seriously by countries globally. In this survey, FPV was the most prevalent pathogen, with a positivity rate of 61.90% (78/126) among the 126 stray cats tested. This is significantly higher than the 37.1% (53/143) reported in the Northeastern China between 2016 and 2017 [14], and the 19.2% reported in China from 2016 to 2019 [15]. It is, however, consistent with the very high prevalence of 73.5% found in a multi-pathogen survey in Southern Italy [16], as well as a recent nationwide investigation in China [17], collectively reinforcing that FPV remains a severe threat to feline populations. Nevertheless, considerable regional variation is evident across studies. For instance, in Changchun, China, the prevalence of FPV positivity in domestic cats with diarrhea was as high as 55.7% (39/70) [18], while in Henan provinces, the positivity rate among suspected cases was only 30.49% (25/89) [19]. Similarly, in 2021, the positivity rate of FPV DNA in domestic cats within and around the Xining Wildlife Park in China reached 54.55% and 47.73%, respectively [20]. These discrepancies suggest that prevalence can vary significantly due to factors such as geographical location, sampling time, and sample size. In summary, the situation of FPV carriage in stray cats in Shenzhen, China, is relatively severe. The likelihood of unvaccinated cats suffering from feline panleukopenia is 8.83 times higher than that of vaccinated cats [21]. Moreover, the presence of FPV has been confirmed in the environment [20]. Vaccination is of great significance in the prevention and control of feline panleukopenia in stray cats. The carriage of FPV in wild animals is relatively common [22]. In this study, Bao’an and Nanshan District of Shenzhen had the highest FPV detection rates. These adjacent districts are characterized by extensive wooded areas and a relatively dense human population density. These environmental and demographic factors might facilitate transmission and could contribute to the high FPV positivity rate observed in stray cats in these areas. Our findings highlight the urgent need for enhanced immunoprophylaxis against FPV in both stray and domestic cats in Shenzhen.

FCV is a highly mutable virus. Its high mutagenicity and genetic plasticity enable the virus to survive successfully within cat populations [23]. In the present study, FCV was the second most prevalent pathogen after FPV, with a positivity rate of 57.14%. Most FCV-positive cats exhibited oral symptoms, consistent with typical clinical manifestations, though a number of asymptomatic carriers were also identified. The prevalence observed here is higher than that reported in several other regions: 31.3% in Beijing, China [24], 26.0% in Kunshan, China [25], 43.0% in Hangzhou, China [26], and substantially higher than the 7.04% in Southern Italy [16], 25.7% in Moscow [27], and 45.0% among suspected cases in Switzerland [28]. Although these samples were collected from clinically diseased cats, all the cats were domestic cats. The cats in this study were all strays. Not only are they unmanaged, but their vaccination coverage is likely to be even lower. This lower vaccination coverage is a plausible contributing factor to the higher FCV positivity rate observed in this population. Furthermore, while vaccination or natural immunity can to some extent prevent FCV infection in cats, those that have been vaccinated or naturally infected may still become carriers after subclinical infection [25,29]. Both infected cats and asymptomatic carriers can continuously shed the virus [30]. What is more, in addition to direct contact transmission, FCV can also be spread through contaminated objects, humans, aerosols, and even flea feces [31]. Therefore, stray cats have a stronger ability to spread FCV. Strict implementation of disinfection, isolation, and quarantine measures is crucial for managing FCV spread and reducing associated mortality.

FCoV-I is primarily transmitted via the fecal-oral route, such as through contact with fecally contaminated items [32]. Stray cats are at high risk of infection through behaviors like competing for food provided by the public and sharing communal defecation sites [33]. Furthermore, natural transmission of FCoV-I between domestic and stray cats has been documented [34,35]. Therefore, there are many factors that influence the transmission of FCoV-I, especially in multi-cat environments where stray cats congregate. In this study, the overall FCoV-I positivity rate was 46.83%, with significant geographical variation across Shenzhen. The highest rate was observed in Longgang District (73.33%), while the lowest was in Futian District (17.39%). Similar disparities in FCoV-I positivity rates have been reported in other studies. For instance, a survey conducted in the Central China region from 2018 to 2020 revealed an overall FCoV-I positivity rate of 46.6% in cats from veterinary clinics, with a positivity rate of 59.3% in samples suspected of FIP [36]. However, the FCoV-I positivity rate was as high as 90% in 120 suspected FIP samples from North and South China [37]. The high risk for stray cats stems not only from frequent contact with other cats but also from potential interactions with wildlife. Additionally, unneutered male cats have been reported to have a higher likelihood of developing FIP [38]. Interestingly, one study in Japan reported a lower FCoV-I positivity rate in stray cats (15.9%) than in domestic cats (35.5%) [2], highlighting that epidemiological patterns can be context-dependent. In conclusion, the high prevalence and distinct spatial variation in FCoV-I observed in this study, coupled with reported transmission routes, indicate that the circulation of this virus among stray cats and at the interface with domestic cats poses a significant challenge for disease control.

In this survey, 29 of 126 stray cats were positive for FHV-I (23.02%), the lowest among all pathogens tested. Previous studies have found that the FHV-I positivity rate in domestic cats in Kunshan, China, was 21.5%. Among these positive cases, the positivity rate was 27.90% for vaccinated cats and 72.09% for unvaccinated cats [39]. Therefore, vaccination is of great significance in controlling FHV-I. In Guangxi, China, the positivity rate of FHV-I in domestic cats was 5.56% [40]. The positivity rate of FHV-I in symptomatic cats from 13 cities in northern and southern China was 16.3% [14]. Adding to this geographical variation, a 2024 study in Yanji City, China, reported an FHV-I prevalence of 13.8% [41], further illustrating the widespread circulation of this pathogen. The samples tested in this study were from stray cats, and the high positivity rate of FHV-I is closely related to the vaccination coverage and management practices. Therefore, it is important to focus on the prevention and control of FHV-I in stray cats.

Rabies is a major global public health issue. This zoonotic disease causes 590,000 deaths annually and affects more than 100 countries worldwide [42]. For live cats, we opted saliva swabs that intermittently shed the rabies virus instead of brain tissue, which is complies with China’s national standards of Nucleic Acid Detection Of Animal Rabies Virus [43,44]. Although recent studies have shown that RABV can be detected in feces, the detection results are related to the degree of sample dilution and exposure time [45]. In this survey, all fresh fecal swab samples were processed promptly after collection to minimize RNA degradation, and no rabies virus nucleic acid was detected. Moreover, the positivity rate of RABV antibodies in the 100 stray cat serum samples was only 6.00%, indicating that the low immunization coverage of rabies vaccines for stray cats in Shenzhen. The overlap between cat and bat habitats creates opportunities for contact and potential rabies transmission. Reports of predation incidents indicate a spillover risk of bats transmitting rabies to cats. [46,47]. Given their wide roaming range, stray cats with negative rabies antibodies may become infected with RABV through wounds if attacked by wild animals or other stray animals. A survey showed that when vaccination coverage exceeded 70%, there were no rabies outbreaks in villages (defined as at least two cases not interrupted by an interval of more than one month). Small-scale outbreaks occurred in villages with lower vaccination coverage, while the largest and longest-lasting outbreak occurred only in villages with vaccination coverage below 20% [48]. This low antibody level suggests that the population is likely susceptible to infection. Large-scale vaccination programs have effectively controlled rabies in dogs, but cats were less vaccinated, mainly due to difficulties in transporting and controlling cats, as well as underestimating of stray cats [49,50]. Given its high fatality rate, rabies control should be prioritized, and vaccination efforts for stray cats intensified to prevent cat-to-human transmission.

Stray cats primarily acquire rabies antibodies through two sources: first, via rabies vaccination administered after rescue; and second, from previously vaccinated domestic cats that have strayed due to accidental loss or abandonment, thereby retaining rabies virus antibodies. According to the results of this study, the antibody positivity rate in stray cats was higher in the central urban area than in the peripheral areas. This may be because pet owners or rescue volunteers in the central urban area have a higher willingness and awareness to vaccinate cats compared to those in the peripheral areas.

Mixed infections with different feline viral pathogens were notably prevalent in the stray cat population, with an overall rate of 62.70% for the four pathogens investigated. This finding is consistent with recent reports, such as the 60.2% mixed infection rate in feline upper respiratory tract disease cases [51]. The most frequent triple co-infection combination involved FCV, FPV, and FCoV-I (12.70%), likely driven by their high individual prevalence and facilitated by the high mobility of stray cats, which amplifies exposure opportunities.

Pairwise association analysis revealed specific patterns: the high Jaccard index between FCV and FPV (0.456) suggests frequent co-detection, possibly due to shared fecal-oral transmission and environmental stability, which are well-documented for both viruses [52,53]. Furthermore, the high odds ratio for FHV–FPV co-occurrence (OR = 2.93, nominal *p* < 0.05) is consistent with the hypothesis that FPV-induced immunosuppression might facilitate FHV reactivation; a mechanism supported by previous studies on the immunosuppressive nature of FPV [8,54]. Although not significant after multiple-testing correction, these associations highlight complex interactions that may exacerbate disease burden and complicate management.

Geospatial analysis showed significant heterogeneity in infection rates across districts. For instance, FCoV-I prevalence was significantly higher in Longgang than in Futian, even after multiple-testing correction. This spatial clustering suggests that localized environmental, ecological, or anthropogenic factors drive transmission dynamics, a phenomenon observed in other urban animal disease systems [55]. We hypothesize that socioecological heterogeneity—such as varying cat densities, which is a known risk factor for directly transmitted pathogens [56], human feeding practices, and access to veterinary services—may underlie these disparities. Higher population density and congregating around feeding sites likely form transmission hotspots, as predicted by core group theory [57], suggesting that targeted interventions in these locations could be most effective.

Based on these findings, we recommend implementing integrated control strategies including: geographically targeted TNR and vaccination programs prioritizing high-prevalence districts such as Longgang for FCoV-I; regular syndromic surveillance at feeding hotspots and shelters; public education for community feeders on hygiene practices; and enhanced veterinary capacity in high-risk areas for improved diagnosis and management of co-infections, particularly those with immunosuppressive interactions.

## Figures and Tables

**Table 1:** Epidemiological profile of five pathogens in 126 stray cats (*Felis vaga*) from Shenzhen City.

Pathogen	Positive Number	Positive Rate (%, 95% CI)
FCoV-I	59	46.83 (38.34–55.51)
FCV	72	57.14 (48.41–65.44)
FHV-I	29	23.02 (16.53–31.10)
FPV	78	61.90 (53.19–69.91)
RABV	0	0.00 (0.00–2.96)
Overall	113	89.68 (83.14–93.87)

**Table 2:** Frequency analysis of specific pathogen co-infection combinations.

Mixed Infection Pathogens	Number of Cats	Proportion Within Co-Infection Category (%)	Proportion in Total Population (%)
Dual infections (Total n = 42)			
FCoV-I + FCV	11	26.19	8.73
FCoV-I + FPV	11	26.19	8.73
FCV + FHV	1	2.38	0.79
FCV + FPV	15	35.71	11.90
FHV-I + FPV	4	9.52	3.17
Triple infections (Total n = 28)			
FCoV-I + FCV + FHV-I	2	7.14	1.59
FCoV-I + FCV + FPV	16	57.14	12.70
FCV + FHV-I + FPV	7	25.00	5.56
FCoV-I + FHV-I + FPV	3	10.71	2.38
Quadruple infection (Total n = 9)			
FCoV-I + FCV + FHV-I + FPV	9	100	7.14
Overall	79	-	62.70

Table footnote: The ‘Proportion within Co-infection Category’ represents the percentage of a specific combination within its respective group (e.g., all dual infections). The ‘Proportion in Total Population’ represents the percentage of cats with a specific combination out of the total 126 sampled cats.

**Table 3:** Analysis of pairwise co-infection associations among the four pathogens.

	Relative Risk (RR)	Odds Ratio (OR)	Jaccard Index
FCoV-I-FCV	1.127	1.756	0.409
FCoV-I-FHV-I	1.031	1.079	0.189
FCoV-I-FPV	1.068	1.400	0.398
FCV-FHV-I	1.147	1.577	0.232
FCV-FPV	1.054	1.395	0.456
FHV-I-FPV	1.281	2.927	0.274

**Table 4:** Pathogen infection profiles of stray cats (*Felis vaga*) across different administrative districts.

District	Number of Cats	Positive Number (Positive Rate)/%				
		FCoV-I	FCV	FHV-I	FPV	RABV
Bao’an	44	19 (43.18)	29 (65.91)	15 (34.09)	33 (75.00)	0 (0.00)
Nanshan	8	4 (50.00)	2 (25.00)	4 (50.00)	6 (75.00)	0 (0.00)
Luohu	8	2 (25.00)	2 (25.00)	0 (0.00)	2 (25.00)	0 (0.00)
Longgang	30	22 (73.33)	14 (46.67)	4 (13.33)	18 (60.00)	0 (0.00)
Longhua	13	8 (61.54)	9 (69.23)	1 (7.69)	5 (38.46)	0 (0.00)
Futian	23	4 (17.39)	16 (69.57)	5 (21.74)	14 (60.87)	0 (0.00)
Total	126	59 (46.83)	72 (57.14)	29 (23.02)	78 (61.90)	0 (0.00)

**Table 5:** Detailed profile of mixed infections in stray cats (*Felis vaga*) across administrative districts.

Mixed Infection Pathogens	Positive Number (Positive Rate)/%					
	Baoan	Nanshan	Luohu	Longgang	Longhua	Futian
FCoV-I + FCV	2 (4.55)	0 (0.00)	1 (12.50)	3 (10.00)	5 (38.46)	0 (0.00)
FCoV-I + FPV	3 (6.82)	2 (25.00)	0 (0.00)	4 (13.33)	1 (7.69)	1 (4.35)
FCV + FHV-I	1 (2.27)	0 (0.00)	0 (0.00)	0 (0.00)	0 (0.00)	0 (0.00)
FCV + FPV	7 (15.91)	1 (12.5)	0 (0.00)	1 (3.33)	0 (0.00)	6 (26.09)
FHV-I + FPV	2 (4.55)	1 (12.50)	0 (0.00)	1 (3.33)	0 (0.00)	0 (0.00)
FCoV-I + FCV + FHV-I	1 (2.27)	0 (0.00)	0 (0.00)	0 (0.00)	0 (0.00)	1 (4.35)
FCoV-I + FCV + FPV	5 (11.36)	1 (12.50)	0 (0.00)	7 (23.33)	1 (7.69)	2 (8.70)
FCV + FHV-I + FPV	4 (9.09)	0 (0.00)	0 (0.00)	0 (0.00)	0 (0.00)	3 (13.04)
FCoV-I + FHV-I + FPV	1 (2.27)	1 (12.05)	0 (0.00)	1 (3.33)	0 (0.00)	0 (0.00)
FCoV-I + FCV + FHV-I + FPV	6 (13.64)	0 (0.00)	0 (0.00)	2 (6.67)	1 (7.69)	0 (0.00)
Total	32 (72.73)	6 (75.00)	1 (12.50)	19 (63.33)	8 (61.54)	13 (56.52)

**Table 6:** Results of neutralizing antibody levels against rabies in stray cats (*Felis vaga*) in 6 districts of Shenzhen.

District	Sample Numbers	Positive Number	Positive Rate/%
Bao’an	38	0	0.00
Nanshan	8	2	25.00
Luohu	8	0	0.00
Longgang	22	3	13.64
Longhua	5	0	0.00
Futian	19	1	5.26
Total	100	6	6.00

**Table 7:** Geographical distribution of rabies neutralizing antibody levels in stray cats (*Felis vaga*) in Shenzhen.

District	Sample Numbers	Positive Number	Positive Rate/%
Urban Area	66	4	6.06
Suburbs	34	2	5.88

## Data Availability

All data and results related to this study are included in the article. Raw data are available from the corresponding author upon reasonable request.

## Supplementary Materials

The following supporting information can be downloaded at: https://www.mdpi.com/article/10.3390/ani15203042/s1, Table S1: The clinical data of the 126 stray cat samples collected in this study; Table S2: Cycle threshold (Ct) values of qPCR detection for five pathogens.
