# Supplementary material for: High Prevalence of Multi-Viral Co-Infections and Low Rabies Seropositivity in Stray Cats of Shenzhen, China
Source: Animals (Basel). 2025 Oct 20;15(20):3042. doi: 10.3390/ani15203042 (PMC12561710; doi:10.3390/ani15203042)
Supplement: Supplementary file 1 [file animals-15-03042-s001.zip › animals-3907083-supplementary.pdf]

Supplementary Table S1. The clinical data of the 126 stray cat samples collected in this study.

Cat ID	Breed	Age	Sex	Weight	Clinical signs	Region
1	Chinese Domestic Cat	1Y+	Male	4kg	-	Futian
2	Chinese Domestic Cat	2Y	Male	3kg	-	Futian
3	Chinese Domestic Cat	1Y	Female	3.5kg	diarrhoea	Futian
4	Chinese Domestic Cat	5M	Female	1.2kg	-	Futian
5	Chinese Domestic Cat	2Y	Male	3.5kg	excessive eye discharge	Futian
6	Chinese Domestic Cat	1Y	Female	2.7kg	-	Futian
7	Chinese Domestic Cat	-	Male	-	-	Longgang
8	Chinese Domestic Cat	-	Female	-	cough	Longgang
9	Chinese Domestic Cat	-	Female	-	cough	Longgang
10	British Shorthair	-	Male	-	dermatosis	Longgang
11	Chinese Domestic Cat	-	Female	-	-	Longgang
12	Chinese Domestic Cat	-	Male	-	thin	Longgang
13	Chinese Domestic Cat	-	Male	-	fat	Longgang
14	British Shorthair	-	Female	-	-	Longgang
15	Chinese Domestic Cat	1Y	Male	3kg	-	Longgang
16	Chinese Domestic Cat	6M	Male	4.5kg	-	Longgang
17	Chinese Domestic Cat	1Y	Female	3kg	-	Longgang
18	Chinese Domestic Cat	5Y	Male	5.5kg	-	Longgang
19	Chinese Domestic Cat	3Y	Female	3kg	-	Longgang
20	Chinese Domestic Cat	1Y	Female	-	-	Longhua
21	Chinese Domestic Cat	1Y	Female	-	-	Longhua
22	Chinese Domestic Cat	1Y	Female	-	-	Longhua
23	Chinese Domestic Cat	1Y	Female	-	-	Longhua
24	Chinese Domestic Cat	5M	Female	-	-	Longhua
25	Chinese Domestic Cat	1Y	Male	-	-	Longhua
26	Chinese Domestic Cat	1Y	Female	-	-	Longhua
27	Chinese Domestic Cat	1Y	Female	-	-	Longhua
28	Chinese Domestic Cat	1Y	Female	-	-	Longhua
29	Chinese Domestic Cat	1Y	Male	-	-	Longhua
30	Chinese Domestic Cat	1Y	Female	-	-	Longhua
31	Chinese Domestic Cat	1Y	Female	-	-	Longhua
32	Chinese Domestic Cat	1Y	Female	-	-	Longhua
33	Chinese Domestic Cat	6M	Male	-	-	Futian
34	Chinese Domestic Cat	7M	Male	-	-	Futian
35	Chinese Domestic Cat	1Y	Female	-	-	Futian
36	Chinese Domestic Cat	1Y	Female	-	-	Futian
37	Chinese Domestic Cat	1Y	Female	-	-	Futian
38	Chinese Domestic Cat	1Y	Female	-	-	Futian
39	Chinese Domestic Cat	1Y	Female	3.32kg	-	Futian
40	Chinese Domestic Cat	1Y	Female	-	-	Futian
41	British Shorthair	1Y	Male	2.8kg	-	Futian
42	Chinese Domestic Cat	1Y	Male	2.8kg	-	Futian
43	British Shorthair	3Y	Male	4kg	excessive eye discharge	Luohu
44	Chinese Domestic Cat	2Y	Female	2.7kg	tapeworm infection	Luohu
45	Ragdoll	3Y	Male	4.8kg	-	Luohu
46	Chinese Domestic Cat	2Y	Female	3.4kg	cough	Luohu
47	Chinese Domestic Cat	3Y	Female	3.8kg	excessive eye discharge	Luohu
48	Chinese Domestic Cat	2Y	Female	3kg	-	Luohu
49	Chinese Domestic Cat	1Y	Female	3.1kg	-	Luohu
50	Chinese Domestic Cat	2Y	Female	2.5kg	-	Luohu
51	-	-	-	-	-	Bao'an
52	-	-	-	-	-	Bao'an

53	-	-	-	-	-	Bao'an
54	Chinese Domestic Cat	2Y	-	-	-	Futian
55	Hybrid Cat	-	-	-	-	Futian
56	British Shorthair	4Y	-	-	-	Futian
57	Chinese Domestic Cat	1Y	-	-	-	Futian
58	Chinese Domestic Cat	2Y	-	-	-	Futian
59	British Shorthair	3Y	-	-	-	Futian
60	-	-	-	-	-	Futian
61	Chinese Domestic Cat	1Y	Female	2.5kg	-	Bao'an
62	Chinese Domestic Cat	2Y	Female	2.8kg	-	Bao'an
63	Chinese Domestic Cat	8M	Female	2.5kg	-	Bao'an
64	Chinese Domestic Cat	1Y	Male	3.5kg	-	Bao'an
65	Chinese Domestic Cat	1Y	Female	3kg	-	Bao'an
66	Chinese Domestic Cat	1Y	Male	4kg	-	Bao'an
67	Chinese Domestic Cat	8M	Male	2.2kg	-	Bao'an
68	Chinese Domestic Cat	1Y	Female	3.5kg	-	Bao'an
69	Chinese Domestic Cat	1Y	Female	3kg	-	Bao'an
70	Chinese Domestic Cat	1Y	Female	2.6kg	-	Bao'an
71	Chinese Domestic Cat	1Y	Female	3.4kg	-	Bao'an
72	Chinese Domestic Cat	1Y	Female	2.5kg	-	Bao'an
73	Chinese Domestic Cat	1Y	Female	2.5kg	stomatitis	Bao'an
74	Chinese Domestic Cat	1Y	Female	3kg	-	Bao'an
75	Chinese Domestic Cat	1.5Y	Male	4kg	-	Bao'an
76	Chinese Domestic Cat	1Y	Male	3.5kg	stomatitis	Bao'an
77	Chinese Domestic Cat	1Y	Female	2.4kg	-	Bao'an
78	Chinese Domestic Cat	1Y	Female	3.1kg	-	Bao'an
79	Chinese Domestic Cat	1Y	Male	2.8kg	-	Bao'an
80	Chinese Domestic Cat	1Y	Female	3.2kg	-	Bao'an
81	Chinese Domestic Cat	1.5Y	Male	4kg	-	Bao'an
82	Chinese Domestic Cat	11M	Female	2.5kg	-	Bao'an
83	Chinese Domestic Cat	11Y	Male	4kg	-	Bao'an
84	Chinese Domestic Cat	7M	Female	2kg	-	Bao'an
85	Chinese Domestic Cat	9M	Female	2.1kg	-	Bao'an
86	Chinese Domestic Cat	2Y	Female	3kg	-	Longgang
87	American Shorthair	2Y	Female	3kg	-	Longgang
88	Russian Blue	4Y	Male	3.5kg	-	Longgang
89	British Shorthair	4Y	Male	3.5kg	-	Longgang
90	British Shorthair	4Y	Male	2.7kg	-	Longgang
91	Chinese Domestic Cat	3Y	Female	3kg	-	Longgang
92	Chinese Domestic Cat	2Y	Female	2.5kg	-	Longgang
93	Chinese Domestic Cat	2Y	Female	2.5kg	stomatitis	Longgang
94	Chinese Domestic Cat	1Y	Female	-	-	Bao'an
95	Chinese Domestic Cat	1Y	Female	-	-	Bao'an
96	Chinese Domestic Cat	1Y	Male	-	-	Bao'an
97	Chinese Domestic Cat	1Y	Female	-	-	Bao'an
98	Chinese Domestic Cat	1Y	Male	-	-	Bao'an
99	Chinese Domestic Cat	1Y	Male	-	-	Bao'an
100	Chinese Domestic Cat	1Y	Male	-	-	Bao'an
101	Chinese Domestic Cat	1Y	Male	-	-	Bao'an
102	Chinese Domestic Cat	1Y	Male	-	-	Bao'an
103	Chinese Domestic Cat	1Y	Male	-	-	Bao'an
104	Chinese Domestic Cat	1Y	Male	-	-	Bao'an
105	-	-	-	-	-	Bao'an
106	-	-	-	-	-	Bao'an
107	-	-	-	-	-	Bao'an
108	-	-	-	-	-	Nanshan
109	-	-	-	-	-	Nanshan
110	-	-	-	-	-	Nanshan
111	-	-	-	-	-	Nanshan

112	-	-	-	-	-	Nanshan
113	-	-	-	-	-	Nanshan
114	-	-	-	-	-	Nanshan
115	-	-	-	-	-	Nanshan
116	-	-	-	-	-	Bao'an
117	-	-	-	-	-	Bao'an
118	Chinese Domestic Cat	-	Male	1.5kg	-	Longgang
119	Chinese Domestic Cat	-	Male	1.5kg	-	Longgang
120	Chinese Domestic Cat	-	Male	1kg	-	Longgang
121	Chinese Domestic Cat	-	Female	1kg	-	Longgang
122	Chinese Domestic Cat	-	Female	1kg	-	Longgang
123	Chinese Domestic Cat	-	Male	1.5kg	-	Longgang
124	Chinese Domestic Cat	-	Female	1kg	-	Longgang
125	Chinese Domestic Cat	-	Male	1.5kg	-	Longgang
126	Chinese Domestic Cat	-	Male	1.5kg	-	Longgang

Supplementary Table 1 presents the clinical data of the 126 stray cats collected in this study. In the 'Clinical Signs' column, a dash ('-') denotes the absence of obvious clinical signs, while in all other columns, it indicates unknown information.

Supplementary Table S2. Cycle threshold (Ct) values of qPCR detection for five pathogens.

Cat ID	FCoV-I	FCV	FHV-I	FPV	RABV
1	-	23.64	-	33.11	-
2	-	-	-	-	-
3	-	33.43	-	35.08	-
4	-	22.27	34.24	33.72	-
5	21.32	22.14	-	32.25	-
6	-	27.68	-	-	-
7	34.12	27.59	35.78	32.69	-
8	22.85	20.70	-	34.28	-
9	30.56	33.07	34.93	35.07	-
10	20.64	-	33.35	34.10	-
11	23.65	24.56	-	33.64	-
12	-	-	-	-	-
13	-	23.28	-	35.00	-
14	-	-	-	-	-
15	-	-	-	33.21	-
16	33.42	-	-	-	-
17	-	-	-	-	-
18	-	-	-	34.32	-
19	34.07	-	-	35.04	-
20	-	21.71	-	-	-
21	31.58	34.51	-	-	-
22	30.25	24.67	31.89	35.05	-
23	-	25.74	-	-	-
24	20.56	21.17	-	-	-
25	26.34	34.03	-	-	-
26	35.02	33.61	-	-	-
27	-	-	-	28.60	-
28	19.52	-	-	33.24	-
29	-	-	-	34.84	-
30	35.07	30.63	-	30.11	-
31	22.35	30.57	-	-	-
32	-	-	-	-	-
33	-	-	-	35.79	-
34	-	23.53	-	34.62	-
35	-	34.22	-	32.50	-

36	-	-	-	35.55	-
37	-	19.57	-	-	-
38	-	22.40	-	-	-
39	-	-	-	-	-
40	-	25.26	-	35.26	-
41	19.85	27.32	34.88	-	-
42	-	22.00	-	-	-
43	27.11	30.96	-	-	-
44	-	34.83	-	-	-
45	-	-	-	-	-
46	-	-	-	-	-
47	-	-	-	27.26	-
48	-	-	-	33.91	-
49	-	-	-	-	-
50	29.60	-	-	-	-
51	25.96	27.56	-	30.76	-
52	-	-	-	32.52	-
53	23.01	29.68	14.60	35.31	-
54	-	-	-	-	-
55	32.77	-	-	34.10	-
56	-	32.44	33.27	32.67	-
57	-	32.73	-	31.13	-
58	-	29.35	30.94	28.34	-
59	-	-	32.47	-	-
60	30.04	24.84	-	26.43	-
61	-	-	-	-	-
62	-	24.79	-	26.02	-
63	-	29.62	-	34.71	-
64	-	-	-	-	-
65	-	30.21	-	34.15	-
66	24.37	-	-	-	-
67	32.65	-	-	32.14	-
68	29.36	34.79	-	28.43	-
69	-	-	-	35.74	-
70	-	-	-	-	-
71	-	22.39	-	29.82	-
72	-	-	32.34	24.04	-
73	-	13.70	29.35	35.26	-
74	-	-	30.77	31.67	-
75	20.08	26.23	35.02	34.51	-
76	30.57	20.45	32.24	32.66	-
77	32.31	-	32.92	33.27	-
78	-	-	-	35.30	-
79	-	-	-	34.45	-
80	-	34.49	33.94	35.55	-
81	-	23.68	-	35.07	-
82	20.68	34.15	32.68	34.20	-
83	18.95	25.74	31.42	31.62	-
84	-	24.06	34.71	32.75	-
85	-	24.61	-	34.01	-
86	-	-	33.48	35.47	-
87	33.91	20.34	-	30.17	-
88	-	20.01	-	-	-
89	20.74	-	-	33.10	-
90	20.34	25.01	-	33.46	-
91	33.84	31.48	-	31.76	-
92	35.28	28.76	-	-	-
93	33.01	26.43	-	-	-
94	-	17.61	-	-	-

95	-	20.33	-	31.45	-
96	35.49	35.77	-	32.30	-
97	-	33.06	28.67	33.45	-
98	22.64	35.64	35.41	32.30	-
99	34.83	21.04	16.79	-	-
100	-	22.44	-	-	-
101	-	21.38	16.48	-	-
102	22.64	22.39	-	-	-
103	20.82	23.62	-	35.78	-
104	-	26.16	-	-	-
105	29.60	26.57	-	32.31	-
106	35.45	21.58	-	-	-
107	22.30	-	-	35.57	-
108	27.50	-	-	31.49	-
109	-	-	29.28	-	-
110	23.08	-	34.73	30.64	-
111	-	-	26.55	-	-
112	-	-	33.90	35.03	-
113	30.35	21.88	-	31.90	-
114	18.22	-	-	4.79	-
115	-	26.47	-	29.66	-
116	-	-	-	32.25	-
117	30.24	-	-	34.84	-
118	28.02	-	-	-	-
119	29.45	-	-	-	-
120	35.21	-	-	-	-
121	30.09	21.35	-	-	-
122	29.70	-	-	-	-
123	17.84	-	-	18.07	-
124	21.78	-	-	32.52	-
125	35.43	32.87	-	29.96	-
126	32.13	33.08	-	33.79	-

This table presents the final confirmed Ct values for feline coronavirus type I (FCoV-I), feline calicivirus (FCV), feline herpesvirus type I (FHV-I), feline panleukopenia virus (FPV), and rabies virus (RABV). Any initially questionable results were subjected to repeat testing, and the values shown reflect the conclusive findings from this verification process. A dash (-) indicates samples that tested negative, for which no Ct value was obtained.
